# Surface Modification of Cyclic-Olefin-Copolymer (COC)-Based Microchannels for the Large-Scale Industrial Production of Droplet Microfluidic Devices

**DOI:** 10.3390/bioengineering10070763

**Published:** 2023-06-25

**Authors:** Yefeng Guan, Huiru Zhang, Zhibin Yan, Xue Wei, Zhuo Zhang, Xuelian Chen

**Affiliations:** 1 Key Laboratory of Biomaterials of Guangdong Higher Education Institutes, Department of Biomedical Engineering, Jinan University, Guangzhou 510632, China; yefengguan@gidichina.org (Y.G.); weixue_jnu@hotmail.com (X.W.); 2 Guangdong Shunde Innovative Design Institute, Foshan 528300, China; 13610293093@163.com (Z.Z.); 2021024065@m.scnu.edu.cn (X.C.); 3 Guangdong Foshan Lianchuang Graduate of Engineering, Foshan 528300, China; 4 Guangdong Provincial Key Laboratory of Optical Information Materials and Technology & Institute of Electronic Paper Displays, South China Academy of Advanced Optoelectronics, South China Normal University, Guangzhou 510006, China

**Keywords:** droplet-based microfluidics, COC, surface modification, industrial production, PCR

## Abstract

The copolymers of cycloolefin (COC), a type of thermoplastic material, have been widely used for the large-scale industrial fabrication of droplet microfluidic devices, which is often performed using hot-embossing or injection-molding techniques. The generation of droplets and the uniformity of droplet sizes are significantly affected by the surface wettability of COC during fabrication and the pressure stability of the employed fluid pump during operation. In order to alleviate the effects of undesirable surface wettability and pressure variation on the generation of droplets in COC-based devices, a simple surface modification procedure was applied to hydrophobically modify the surfaces of COC-based microchannels for large-scale industrial production. The surface modification procedure consisted of an oxygen plasma treatment of the polymer surface followed by a solution-phase reaction in fluorocarbon solvent. The experimental results demonstrate that following the proposed surface modification, the COC droplet microfluidic devices could stably generate microvolume water droplets with a small coefficient of variation, even if the pressure of the dispersed phase (water) fluctuated. The durability test results regarding the modified surfaces show that the hydrophobicity of the modified COC surfaces could be sustained for up to four months, deteriorating with time thereafter. Our study can provide a potential solution useful in and guidance for the large-scale industrial production of droplet microfluidic devices for various applications, including polymerase chain reaction and single-cell analysis.

## 1. Introduction

Droplet-based microfluidics is a kind of microfluidic technology that utilizes the interaction between flow shear force and surface tension in microchannels to divide two different immiscible fluids into discrete droplets with nanoscale or smaller volumes. The extremely high flux of droplet formation can usually produce thousands of microdroplets per second [1,2]. Generally, to facilitate an improvement in the dispersion stability of the generated droplets, surfactants are added into one of the liquid phases to avoid the merging of multiple droplets so that the microdroplet system remains stable, even under high-temperature conditions [3,4,5,6,7]. Each droplet can act as an independent microreactor to significantly increase the specific surface area of the entire reaction system, allowing for the various reaction rates and heat transfer rates to be accelerated while also reducing the potential for cross-contamination [8]. Accordingly, droplet-based microfluidics has gradually become one of the new tools in the fields of molecular biology, drug synthesis, single-cell analysis, and nanomaterial synthesis [9,10,11,12,13]. Notably, in the field of molecular biology, droplet-based microfluidics has already been successfully applied to digital polymerase chain reaction (PCR) analysis, including the QX series of digital PCR instruments produced by the Bio-rad Company, which are extensively used as promising molecular diagnostic devices. Compared to real-time fluorescence quantitative PCR instruments, digital PCR instruments can acquire data from absolute quantitative analysis via the observational counting of individual microdroplets after amplification. Additionally, reagent consumption can be reduced via the microdroplet method, leading to increased heat transfer efficiency during the PCR process [14,15,16,17,18,19].

In order to avoid the cross-contamination of biological samples, which probably affects the accuracy of the subsequent experimental results, disposable microdroplet generation chips are often incorporated in digital PCR instruments. Due to their high production capacity and low cost, polymer injection-molding chips are considered the optimal choice for digital PCR instruments. Among them, the copolymers of cycloolefin (COC) exhibit the merits of excellent optical transparency, chemical resistance, low water absorption, and good biocompatibility; thus, they are often employed as raw materials for the polymer injection molding of microdroplet generation chips [20,21,22]. The COC offer numerous advantages, but they are not suitable for some commonly used hydrophobic liquid reagents because of their poor wettability; thus, the corresponding surface modifications are still required. For example, Roy et al. carried out a plasma treatment on the surfaces of COC to determine their hydrophilicity by adjusting the amounts of different gases, power levels, and time. Moreover, the surface morphologies and wettability of COC were also compared under different conditions, while the influences of surface modification on the bonding strength of the COC chips were explored. The corresponding experimental results demonstrated that the processing of COC surfaces via plasma treatment can not only render them hydrophilic but also improve the corresponding bonding strength [23,24]. Furthermore, Balamurugan et al. studied the surface fluorination modification of polymer materials such as PC, PMMA, and COC, and the dimensional uniformity of the generated microdroplets was increased by improving the surface hydrophobicity of the materials [25]. Su et al. realized bonding and the endowment of hydrophobicity in one step through an approach consisting of simultaneous solvent bonding and surface fluorination [26].

Nevertheless, the current studies mainly focus on the intuitive improvement of the uniformity and stability of water-in-oil droplets produced via a chip hydrophobic treatment in a laboratory environment, but much broader concerns should also be addressed in the industrial production process. For instance, a variety of factors, including the small differences in the microchannel sizes of the chips during mass production, pressure fluctuations caused by the constant operation of the pressure pump in droplet generation devices, and variations in the microchannels during the bonding of different batches, can contribute to the changes in microdroplet sizes. In contrast, for PCR systems, when the microdroplet size tolerance exceeds ±3 μm, the corresponding microdroplet would be identified as an invalid microdroplet during the subsequent PCR data analysis. It should also be noted that a poor uniformity of microdroplet sizes does not align with the statistical principle of the Poisson distribution followed in the design of PCR instruments. To decrease the sensitivity of the microdroplet generation process to pressure fluctuations and the precision of microfabrication, it is hypothesized that the changes in the microdroplets caused by such varying factors can be mitigated through the hydrophobic treatment of microchannels, allowing for more uniform microdroplets to be stably generated and an increase in the number of effective microdroplets required for PCR detection.

In this work, the surface morphologies, functional groups, and contact angle (CA) changes of COC chips before and after fluorination treatment were systematically investigated. The droplet formation process of COC microchannels and the uniformity of the microdroplet sizes were compared and analyzed between the chips with and without fluorination treatment within one week of fluorination treatment and four months of fluorination treatment. The influences of microchannel fluorination on the uniformity and stability of microdroplet formation were revealed, and the effects of the storage time of the fluorination-treated COC chips were also elucidated. Additionally, the effects of water-phase pressure on the changes in droplet sizes and the droplet formation process were studied when the other conditions were fixed. Finally, the fluorination-treated COC-based chips in this work were implemented in a PCR test. This work may serve as an important reference for the future mass production of commercial droplet microfluidic chips.

## 2. Experimental Section

### 2.1. Materials

The COC sheets (No. 8007) used in this work were purchased from Topas Advanced Polymers Gm-bH, Japan; these sheets are optical-grade amorphous materials employed in medical, optical, and other advanced packaging applications. The COC material was prepared via the copolymerization of ethylene and norbornene, and the corresponding *T*_g_ of 78 °C and melting index of 32 cm^3^/10 min (260 °C, 2.16 kg) were obtained. A substrate with microchannels and reservoirs was produced using the injection-molding technique, while the film of the cover was extruded and then cut into the same dimensions as the bonding surface of the substrate (76 mm × 26 mm). The 1H, 1H, 2H, 2H-perfluorooctane trichlorosilane and isopropyl alcohol used were produced by Macklin Biochemical Technology Co., Ltd. (Shanghai, China). The fluorinated solution FC-3283 (perfluorotripropylamine) used was produced by 3M Co., Ltd. (St. Paul, MN, USA), and the deionized water used was obtained from the DIRECT-Q3UV ultrapure water system of MERCK MILLIPORE, Germany. In this experiment, the water phase (dispersed fluid) and oil phase (carrier fluid) (containing about 8% surfactant) used were products independently developed by our research group.

### 2.2. Material Characterization

Sessile water droplet CA measurements were performed using a CA system (Shengding, SDC-200S, Chengdu, China) equipped with a camera and possessing data-processing functions. For each measurement, 2 μL of deionized water was deposited onto the polymer surface, and the water CA was detected immediately using the employed instrument’s software. For each test condition, measurements were taken at at least 3 different positions, and each position was tested twice, thereby allowing for an averaged value to be determined.

The X-ray photoelectron spectra (XPS) were captured using Escalab 250XI (Thermofisher Co., Waltham, MA, USA) with a monochromatic Al Kα (hv = 1486.6 eV) excitation source (150 W), a beam spot with a diameter of 650 μm, and a photoelectron emission angle of 45°, and charge correction was carried out using contaminated carbon C 1 s = 284.8 eV. The vacuum degree of the analysis chamber was set at 1 × 10^−10^ mbar. The analysis area and sampling depth were about 0.4 mm^2^ and 10 nm, respectively, and a constant analyzer pass energy was applied, which was accompanied by a narrow sweep of 30 eV and a wide sweep of 100 eV.

The surface morphologies and microstructures of COC before and after surface fluorination treatment were observed using a scanning electron microscope (Hitachi, SU8020, Tokyo, Japan) with an acceleration voltage of 3 kV. Before the observation, each sample was subjected to gold spraying under a vacuum. Subsequently, the surface morphologies of the COC films, including the original state (COC), that after oxygen plasma cleaning (COC-O), and that after fluorination treatment (COC-O-F), were obtained.

The process of generating the droplets and the diameters of the droplets were observed using an IX73 optical microscope and DP73 CCD from Olympus Co. (Tokyo, Japan). In the experiment, the COC droplet chip was fixed on the movable sample stage of the microscope, and a monochrome CCD camera was utilized to record the entire droplet generation process.

### 2.3. Surface Fluorination

**Scheme 1:**
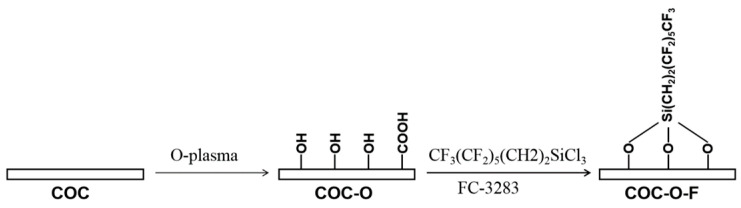
Reaction scheme used for the fluorination of COC.

The fluorination treatment of the COC sheets was a two-step process (Scheme 1). In the first step, the COC sheet was treated with oxygen plasma at room temperature (the resultant sample was denoted as COC-O). The oxygen plasma was produced using a radio frequency (13.56 MHz) plasma reaction system (PDC-MG, Chengdu, China), and the specific parameters set were as follows: a power level of 120 W, treatment time of 30 s, and oxygen gas flow rate of about 0.03 L/min (these parameters were used for all plasma treatments in this paper). In the second step, after oxygen plasma treatment, the COC sheet was soaked in FC-3283 solution containing 10% 1H, 1H, 2H, and 2H-perfluorooctane trichlorosilane for 2 h. Thereafter, the sheet was taken out and washed with FC3283 until the surface became transparent, after which it was cleaned with isopropyl alcohol and deionized water (the resultant sample was denoted as COC-O-F). After being washed until there was no obvious oil contamination or particulate matter on its surface, the sheet was placed in an oven for drying at 65 °C.

For the fluorination treatment of the COC microchips, the plasma-cleaned COC substrate and cover were bonded by a thermal-compression-bonding machine, and the FC-3283 solution containing 10% 1H, 1H, 2H, and 2H-perfluorooctane trichlorosilane was injected into both the water-phase and oil-phase reservoirs. Afterwards, pressure was applied to fill the entire microchannels with the fluorination reagent, and the microchannels were fully exposed to the reagent by adjusting the pressure. After the reagent had been retained in the microchannels for 2 h, the microchannels were first washed with the FC3283 solution until there were no impurities in the chip. Then, the microchannels were washed with isopropyl alcohol and deionized water in sequence, and the residual reagent was dried in an oven at 65 °C.

### 2.4. Manufacturing Process of Large-Scale Droplet Microfluidic Devices

**Figure 1:**
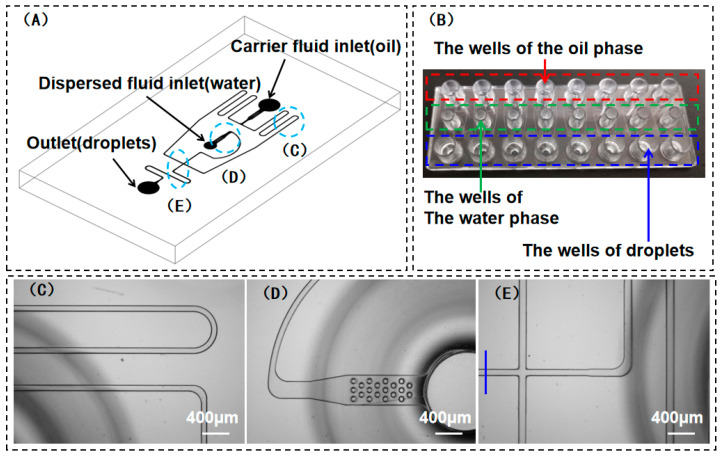
(**A**) Schematic diagram of the structure of water-in-oil droplet chip (COC) for the generation of 100–120 μm microdroplets; (**B**) physical chip and its corresponding instructions; (**C**–**E**) enlarged images of the blue circles as marked in (**A**); (**C**) continuous-phase microchannel size: 110 μm × 50 μm (width × height); (**D**) filter columns at the exit of discrete-phase microchannel; (**E**) the cross junction of the microchannel and dimensions were tested at the blue line.

In this study, the manufacturing process of droplet microfluidic devices can be divided into the following three steps. The first step was the design stage, for which the objective was to stably generate uniform droplets with an average diameter of 110 ± 2 μm. The design was conducted on the basis of the numerical simulation results. Then, a PDMS chip was fabricated for verification. Subsequently, the microchannel size was determined after repeated modifications and experimental verifications. The second step was the manufacturing of the mold, which consisted of two parts: the production of a mold for the microchannels and for the wells. The microchannel structure of the mold was produced via nickel electroforming. The steel mold of the wells was made via precision machining. The physical chip is presented in Figure 1B. It can be seen that there are eight independent droplet generation devices on each chip with identical shapes and sizes, and each reactor is composed of three wells, corresponding to an oil phase, a water phase, and a droplet phase (Figure 1B). The third step was thermal compression bonding. To bond the chips, the injection-molded substrate and as-extruded COC cover needed to be bonded using the thermal-compression-bonding technique. First, the surfaces of the substrate and cover were bonded and then adhered together using a thermal-compression-bonding machine operating at a certain temperature and pressure. It should be noted that no binders or any other substances were added during this process to ensure that the microchannels were not contaminated. It is crucial to select the appropriate temperature and pressure values. For example, values that are too large can induce issues such as microchannel deformation and dimensional changes. In contrast, values that are too small can lead to issues such as low bonding force and liquid leakage during use. Obviously, the flatness of bonding machine is the most important condition to ensure the uniformity and stability of the eight channels. For the chips used in the present study, the microchannel widths varied in different positions, but with a fixed channel depth (50 μm). The tolerance for each position of the channel was not greater than ±5 μm. The largest influence on the droplet is the water channel before the cross junction, which is about 500 μm from the cross junction (as shown in Figure 1E). The dimensional tolerance of this position is about ±3 μm. Accordingly, the influence of the difference in sizes between microchannels could be neglected in the following experiment.

### 2.5. Liquid–Liquid Segmented Flow Experiments

A droplet microfluidic device was employed to generate water-in-oil microdroplets. First, 30 μL of FC-3283 (oil) containing 8% block copolymer surfactant was added to the oil-phase (the carrier fluid) reservoir. The surfactant was synthesized by coupling perfluorinated polyethers (PFPE) with polyethyleneglycol (PEG). Additionally, 20 μL of water containing the Tap enzyme, nucleic acid, and chemical dyes was also added to the water-phase (the dispersed fluid) well (Figure 1B). Afterwards, a constant air pressure was simultaneously applied to both the oil-phase and the water-phase wells; thus, the water-phase flow was sheared by the oil-phase flow at the cross junction and thus formed water-in-oil droplets, allowing for such droplets to finally enter their specified wells (Figure 1B). When observing the diameter of a droplet, it needed to be moved onto a microscope slide using a pipette, allowing for the measurement of its diameter via observation through the microscope.

## 3. Results and Discussion

### 3.1. Influence of Plasma Cleaning and Fluorination on the Surface Wettability of COC

**Figure 2:**
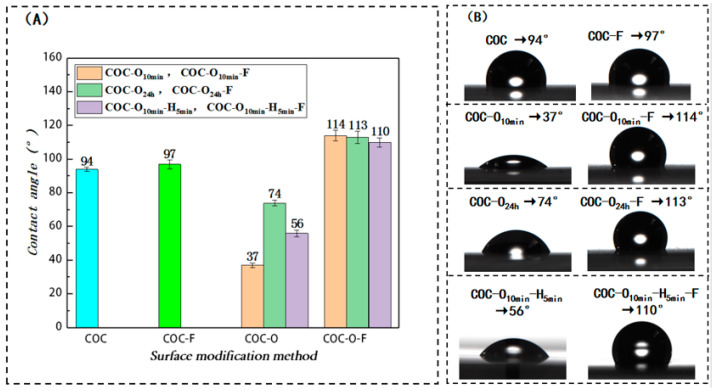
Influence of various surface treatment approaches on the water CA of COC surface. (**A**) Variations in water CA on the COC surface cleaned with oxygen plasma and then processed via fluorination treatment after being left for 10 min, for 24 h, and for 10 min and then being heated for 5 min, respectively; (**B**) CA results of COC after various surface treatments. Remark: COC-O_10 min_ means that the sample is left at the oven room temperature for 10 min after plasma; similarly, COC-O_24 h_ means that the sample was left in an oven at room temperature for 24 h after plasma treatment. COC-O_10 min_-H_5 min_ means that the sample was left in an oven at room temperature for 10 min after plasma treatment and then heated in an 78 °C oven for 5 min.

First, the surface wettability of COC without fluorination surface treatment was studied. A COC sheet was cleaned with deionized water and dried in an oven at 65 °C, and the as-obtained sample was denoted as COC. The CA was 94° (Figure 2). Second, the COC sheet was fluorinated according to the procedures mentioned in Section 2.3. The as-obtained sample was denoted as COC-F, and the corresponding CA for COC-F was 97° (Figure 2). The experimental results demonstrate that the surface hydrophobicity of the COC sheet that was fluorinated was slightly superior to the COC that did not undergo the fluorination treatment. This shows that it is difficult to significantly enhance the surface hydrophobicity of COC using a direct fluorination treatment. Third, the surface wettability of the plasma-cleaned COC was experimentally studied. The COC sheet was plasma-treated using oxygen as a medium and denoted as COC-O. Some COC-O samples were placed in an oven at room temperature for 10 min (the resultant samples were denoted as COC-O_10 min_), while other COC-O samples were placed in an oven for 24 h (these samples were denoted as COC-O_24 h_). Figure 2 shows that the CAs of the water droplet on COC-O_10 min_ and COC-O_24 h_ were 37° and 74°, respectively. The experimental results show that the surface wettability of COC can be partially recovered as the exposure time of COC-O to the air is increased. Fourth, the surface wettability of COC treated via plasma cleaning followed by fluorination treatment was experimentally analyzed. The COC-O_10 min_ and COC-O_24 h_ samples were fluorinated using the method mentioned in Section 2.3. The resultant samples were named COC-O_10 min_-F and COC-O_24 h_-F. Their corresponding CAs were determined to be 114° and 113°, respectively (Figure 2). In the actual manufacturing process, two COC-O sheets were heated to 78 °C for 5 min and thermally bonded to form microchannels before the fluorination treatment. Thus, a COC-O_10 min_ sheet was firstly heated in an oven (78 °C) for 5 min (labeled COC-O_10 min_-H_5 min_) and then fluorinated after cooling (COC-O_10 min_-H_5 min_-F). The CAs measured were 56° and 110°, respectively. The experimental results demonstrate that the surface wettability of COC after plasma cleaning can be partially recovered after placing it in an air atmosphere with different temperatures, but the change in the interval time and temperature has a limited impact on the surface wettability of COC after fluorination treatment. Compared to the COC-F, both the CAs of COC-O_10 min_-F, COC-O_24 h_-F, and COC-O_10 min_-H_5 min_-F were remarkably increased. The experimental results indicate that plasma cleaning plays an important role in the hydrophobic modification of COC materials.

During the industrial production of COC chips, the bonding surface is usually cleaned using a plasma treatment before bonding to remove surface impurities and improve surface energy, thereby significantly enhancing the bonding force [23,24]. Nevertheless, the surface properties of COC are not stable after plasma cleaning, and the resultant surface wettability can be partially recovered after a period of time. During practical production, this time interval might vary between different chip-processing methods. However, the experimental results also show that within a certain time range (such as 24 h), the difference in the time interval has a limited impact on the product’s surface wettability after fluorination treatment. At the same time, the temperature is elevated during thermal bonding, which could increase the recovery rate of hydrophobicity after plasma treatment. As shown in Figure 2, the CA was increased from 37° to 56° after a thermal bonding period of five minutes. However, after fluorination, the contact angle changed to 110°, which was only slightly smaller than COC-O_10 min_-F (114°) and COC-O_24 h_-F (113°).

### 3.2. Analysis of Molecular Compositions and Physical Morphologies of COC Surface Processed through Plasma Cleaning and Fluorination Treatment

**Figure 3:**
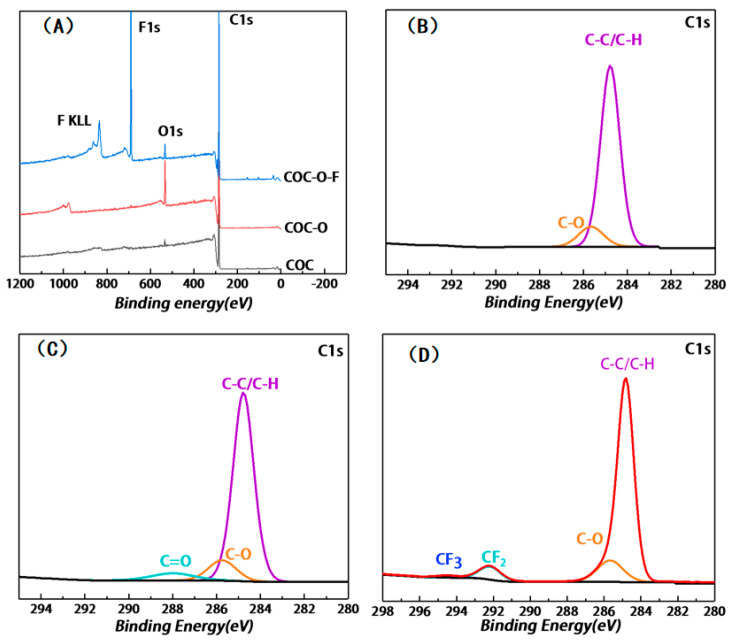
(**A**) XPS broad spectra of COC, COC-O, and COC-O_10 min_-F, which were measured with a monochromatic Al Kα source at X-ray power of 150 W, pass energy of 100 eV, and photoelectron emission angle of 45°; (**B**–**D**) C1s spectra of COC, COC-O, and COC-O_10 min_-F, respectively, which were measured with a monochromatic Al Kα ray source at X-ray power of 150 W, at a pass energy of 30 eV, and with a COC film with a thickness of 250 μm.

Firstly, X-ray photoelectron spectroscopy (XPS) was conducted to analyze the molecular components on the surfaces of the COC samples before and after plasma cleaning and fluorination treatment. Figure 3A shows the XPS broad spectra of COC, COC-O, and COC-O_10 min_-F. Apparently, a strong C signal and a weak O signal were detected on the untreated COC surface, and this finding is basically consistent with the fact that this COC sample contained C and H. Moreover, such a weak O signal is probably due to the presence of oxygen-containing pollutants during the test. After surface modification via oxygen plasma, the COC-O sample displayed a strong O signal. After the surface fluorination treatment, the O signal of the COC-O_10 min_-F sample weakened, but the F signal could be detected.

Figure 3B shows the results of the high-resolution XPS analysis of the untreated COC surface. The C1s spectrum of COC shows a single peak located at ~284.78 eV, which was caused by the presence of C-C/C-H in the material. Meanwhile, a weak C-O peak can also be observed, which may be ascribed to the influence of O-containing impurities during the test. The concentrations of these two components were 87.6% and 12.4%, respectively (as listed in Table 1).

Figure 3C presents the C1s spectrum of the COC-O sample after oxygen plasma cleaning. Compared with Figure 3B, the C-O peak and C=O peak can be simultaneously detected at 285.75 eV and 287.99 eV, respectively, which correspond to the formation of functional groups, such as hydroxyl and carboxyl groups, on the surface of the COC-O sample. The content of C-O/C=O determined was 81.86% and 18.14%, respectively (as listed in Table 1), while the content ratio of C-C/C-H was reduced, which was ascribed to the introduction of oxygen during the oxygen plasma treatment. The COC sample is mainly composed of C-C (3.4 eV) and C-H (4.3 eV), while the energy of oxygen plasma is within the range between several to tens of electron volts, which is higher than the aforementioned bond energies. Accordingly, the surface chemical bonds and main chain of COC were broken after the oxygen plasma treatment, allowing them to reform the covalent bonds with the particles in the excited state [27]. The introduction of O was regarded as the main factor contributing to the high hydrophilicity of the COC-O sample.

Figure 3D shows the C1s spectrum of the COC-O_10 min_-F sample after oxygen plasma cleaning and fluorination treatment. Compared with Figure 3B, the presence of CF_2_ and CF_3_ can be found at 292.19 eV and 294.4 eV in Figure 3C, respectively, and the corresponding content is 8%. Meanwhile, the peak of C=O disappears, whereas the content of C-C/C-H and C-O decreases to 77.94% and 14.06%, respectively. This demonstrates that a fluorinated layer mainly containing CF_2_ and CF_3_ was generated on the surface of COC-O-F sample. F has strong electronegativity allowing it to reduce the surface energy of materials to a relatively low level [28], which is mainly responsible for the high hydrophobicity of the COC-O_10 min_-F sample.

**Figure 4:**
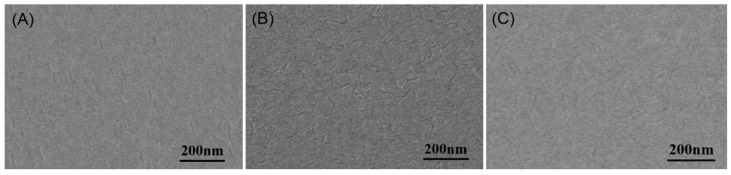
(**A**–**C**) Surface morphologies of COC, COC-O, and COC-O_10 min_-F observed using SEM, respectively, which was observed at 30,000 times magnification and an acceleration voltage of 3 kV.

Additionally, the surface morphologies of the COC sample before and after plasma cleaning and fluorination treatment were observed using SEM (Figure 4A–C). Based on the SEM images, the surface of the untreated COC sample is relatively smooth, while the surface of the COC-O modified by the plasma treatment shows numerous microcracks and its surface roughness is remarkably increased, which may be one of the reasons for the formation of a hydrophilic surface. In contrast, after fluorination treatment, the microcracks became blurred, and there was no significant difference from the initial surface of the COC sample. The microcracks on COC were not due to the plasma treatment; rather, they were produced by the COC film in the extrusion process during the application of tensile force. The size of the microcracks increased after the plasma treatment and appeared clearer under SEM.

### 3.3. Influence of the Fluorination Treatment of Inner Microchannel Surface on Microdroplet Generation

#### 3.3.1. Microdroplet Generation in the COC Chip That Did Not Undergo Fluorination Treatment

**Figure 5:**
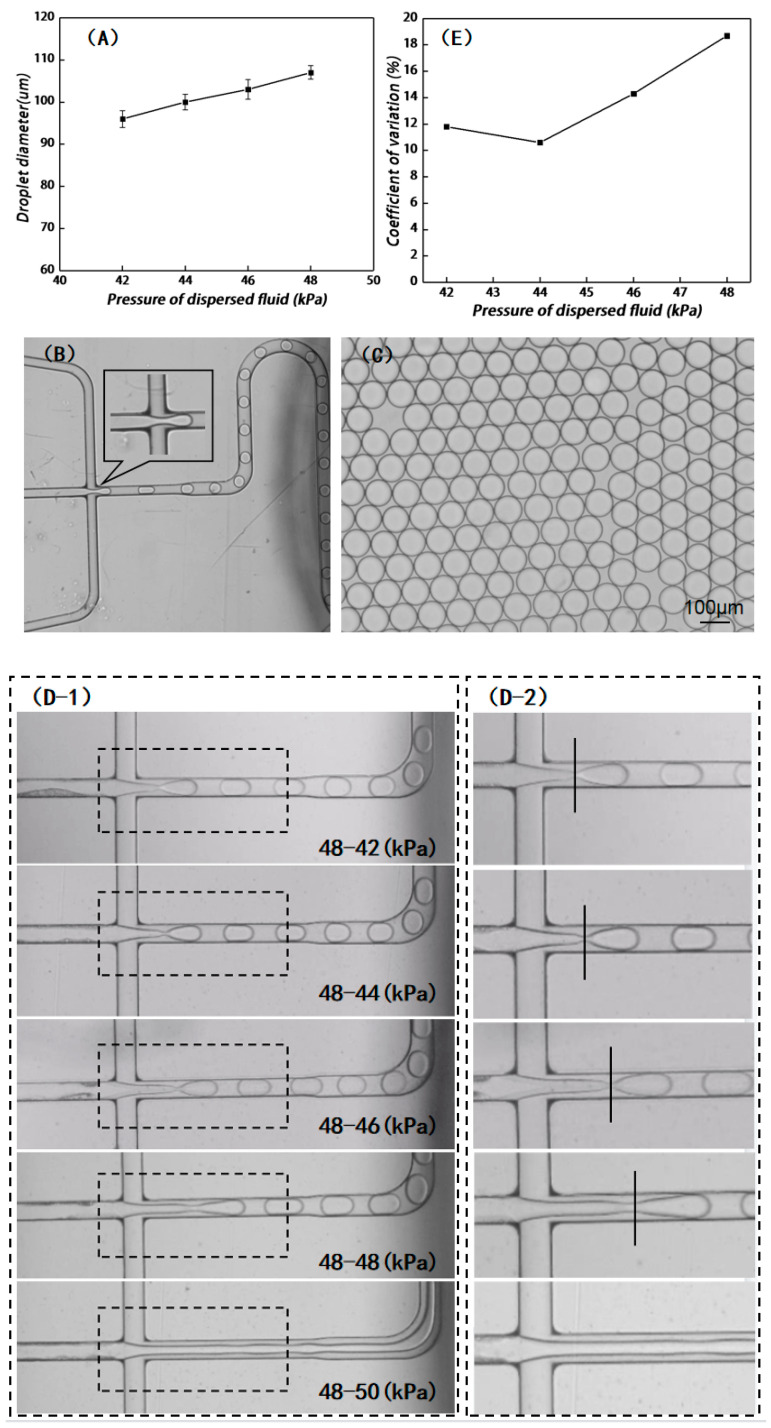

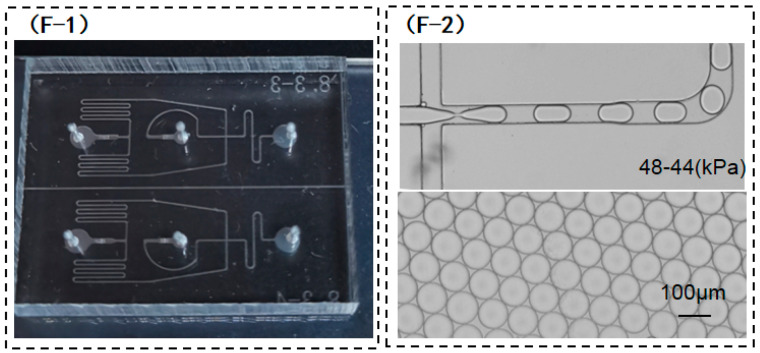
(**A**) Microdroplet diameters under various water-phase pressure levels after the bonding of the untreated COC substrate and cover; (**B**) for ideal microdroplet generation, the discrete phase ends at the cross junction; (**C**) image of microdroplet diameter observed using a microscope, and the related CV values can be calculated using software; (**D**) view of the cross junction when the microdroplets were generated after the COC substrate and cover were directly bonded to form the chip, and (**D-2**) enlarged view of the dashed box as marked in (**D-1**); (**E**) variations in CV values of microdroplet diameters generated under different water-phase pressures; (**F**) microdroplet diameters obtained by the PDMS chips with the same structure; and (**F-1**) a physical picture of PDMS chips and (**F-2**) droplet formation on PDMS chips.

A microchip was fabricated by bonding the COC substrates that did not undergo plasma and/or fluorination treatment. The droplet generation performance of the chip was evaluated under different pressure levels. As shown in Figure 5A, the oil-phase pressure was fixed at 48 kPa, and the water-phase pressure was increased from 42 kPa to 50 kPa. The experimental results demonstrate that the microdroplet size increased with the increase in water-phase pressure. When the water pressure was set to 42 kPa, 44 kPa, 46 kPa, and 48 kPa, the diameters of the microdroplets were 96 μm, 100 μm, 103 μm, and 107 μm, respectively. When the water-phase pressure exceeded 50 kPa, it was difficult to generate microdroplets. Under the pressure range mentioned above, the sizes of the generated microdroplets could not be controlled within the range of 110 ± 2 μm, indicating that these microdroplets could not meet the requirements of our developed PCR device. To explore the droplet generation process, the real-time generation process of microdroplets was systematically observed using a microscope equipped with a high-speed camera. The diameters of the microdroplets were analyzed using the microscope control software, while the coefficients of variation (CV) were calculated to evaluate the uniformity of the microdroplets (Figure 5B,C).

As shown in Figure 5B, for an ideal droplet generation chip, the water-phase completely breaks into droplets of equal size without an extended tail at the cross junction. In this experiment, the water-phase pressure was set from 42 kPa to 48 kPa; it can be seen that the tail of the water phase at the cross junction becomes increasingly and longer as the water-phase pressure increases. When the water-phase pressure exceeded 50 kPa, a laminar flow could be formed in the oil-water phase, and microdroplets could not be generated (Figure 5(D-1) and enlarged view of Figure 5(D-2)). Generally, it is expected that the microdroplets generated by the chip should be as uniform as possible. As the water-phase pressure increases, the coefficients of variation of the microdroplet sizes increase correspondingly, which, in this case, were 11.8%, 10.6%, 14.3%, and 18.7% (Figure 5E).

The above experimental results show that for a given oil-phase pressure, the diameter of microdroplets is enhanced with the increase in water-phase pressure, while the corresponding uniformity of droplet size worsens. When the water-phase pressure exceeds a certain value, it becomes difficult for the shearing force of the oil phase on the water phase to break the water phase, thus leading to the appearance of laminar flow. As mentioned in the analysis of the above experimental data, it is difficult to obtain microdroplets that meet the requirements of a practical PCR test through only regulating the water-phase pressure. Compared with the COC chip, the PDMS chip (Figure 5(F-1)) with the same microchannel structure design can generate microdroplets with a diameter of 110 ± 2 μm within the above pressure range of the oil phase and water phase (Figure 5(F-2)). The CA of PDMS material was calculated to be about 116.5°. During the preparation process of the PDMS chip, the CA is reduced by the plasma cleaning of the bonding surface. Hence, the as-prepared PDMS chip must be dried and left for about 3 days. After recovery via plasma cleaning, microdroplets can be generated, and the CA of PDMS recovers to 110°, which is still larger than the 94° CA of the COC material that did not undergo the fluorination treatment. Accordingly, the fluorination treatment of the COC chip is proposed to increase the CA of the inner microchannel in order to generate larger microdroplets, while the extended tail of the water phase at the cross junction can be avoided.

#### 3.3.2. Formation of Microdroplets in the COC Chip after Fluorination Treatment

**Figure 6:**
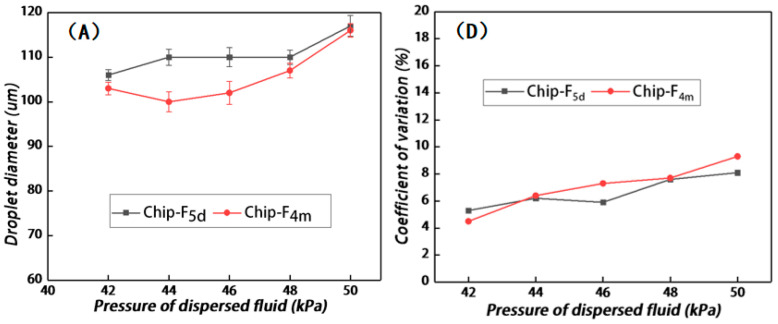

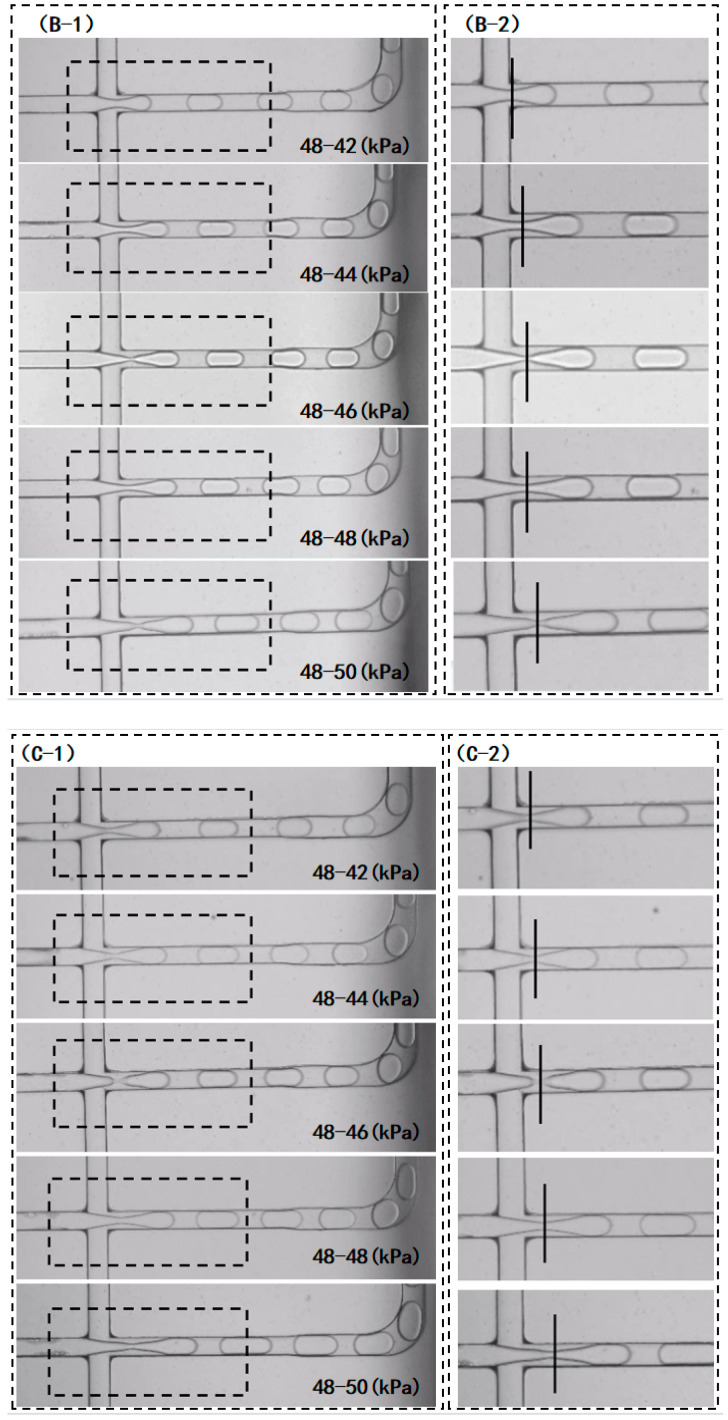
(**A**) Variations in microdroplet diameters generated by Chip-F_5d_ and Chip-F_4m_ under different water-phase pressures; (**B**) real-time situation of the cross junction during the generation of microdroplets by Chip-F_5d_, and (**B-2**) enlarged image of dash box as marked in (**B-1**); (**C**) real-time situation of the cross junction during the generation of microdroplets by Chip-F_4m_, and (**C-2**) enlarged image of dash box as marked in (**C-1**); (**D**) variations in CV values of microdroplet diameters generated by Chip-F_5d_ and Chip-F_4m_ under different water-phase pressures.

To verify the longevity of the fluorination treatment, the chip after fluorination treatment was vacuum-packed and placed at room temperature for 5 days (Chip-F_5d_) and 4 months (Chip-F_4 m_), respectively, and comparative experiments were conducted. The parameters of microdroplet generation are as follows: the oil-phase pressure was 48 kPa and the water-phase pressure was 42 kPa, 44 kPa, 46 kPa, 48 kPa, and 50 kPa, respectively. As presented in Figure 6A, the sizes of the microdroplets generated by Chip-F_5d_ were 106 μm, 110 μm, 110 μm, 110 μm, and 117 μm, which are larger than those generated by the COC chip that did not undergo the fluorination treatment. When the water-phase pressure was 44 kPa, 46 kPa, and 48 kPa, the generated microdroplet size was 110 μm, which conforms to the requirements of practical PCR tests. This demonstrates that the effect of microdroplet size on the fluctuation of water-phase pressure is limited when the water-phase pressure fluctuates unsteadily within the range from 44 kPa to 48 kPa. In contrast, the sizes of microdroplets generated by the Chip-F_4m_ were 103 μm, 100 μm, 102 μm, 107 μm, and 116 μm. Compared with the chip after the fluorination treatment, within one week, all the microdroplet sizes became small and thus failed to meet the requirements of the PCR instrument. This means that the hydrophobic effect is decreased with the prolongation of the storage time after the chip is produced, so Chip-F_4m_ cannot meet the application requirements.

The extended tails at the cross junction were evaluated with respect to the microdroplets generated by Chip-F_5d_ and Chip-F_4m_, respectively. The hysteresis of the water phase at the cross junction was not obvious when the oil/water pressure of Chip-F_5d_ was 48 kPa and 42 kPa, respectively. When the water-phase pressure was 44 kPa, 46 kPa, and 48 kPa, a slight extended tail was observed. However, the microdroplet diameter did not increase with the increase in pressure, which constitutes a significant improvement compared with that of the chip that did not undergo the fluorination treatment. When the water-phase pressure was 50 kPa, the water phase at the cross junction showed slight hysteresis, and the microdroplet size was significantly increased (Figure 6B). As shown in Figure 6C, all the microdroplets generated by Chip-F_4m_ decrease in size, but the extended tail of the water phase at the cross junction is not significantly different from that of Chip-F_5d_. Both Chip-F_4m_ and Chip-F_5d_ exhibit a slight tail, and there is no increasing trend with the increase in pressure, so they still have advantages over the chip that did not undergo the fluorination treatment.

The coefficients of variation of the microdroplet diameters generated by Chip-F_5d_ and Chip-F_4m_ were evaluated. As shown in Figure 6D, for Chip-F_5d_, the CV values of the microdroplet sizes are relatively stable as the water-phase pressure increases; these values were calculated to be 5.3%, 6.2%, 5.9%, 7.6%, and 8.1%, respectively. When the oil and water pressure levels were 48 kPa and50 kPa, respectively, the CV values increased but were still smaller than those of the chip that did not undergo the fluorination treatment. This indicates that more uniform microdroplets can be generated after the fluorination treatment of microchannels. For Chip-F_4m_, as the water-phase pressure increases, the CV values of the microdroplet sizes are increased relative to the value tested within one week after fluorination treatment; these values were calculated to be 4.5%, 6.4%, 7.3%, 7.7%, and 9.3%, respectively. However, compared with the chip without fluorination treatment, the CV value of the microdroplet diameter is much smaller. Therefore, even after 4 months of fluorination treatment, the uniformity of microdroplets is still superior to that of the chip that did not undergo the fluorination treatment.

The flow digital PCR detector independently developed by our research group was used to test the microdroplets generated by the COC chip after fluorination treatment, and the water-phase pressure levels were set to 44 kPa, 46 kPa, and 48 kPa. The corresponding results are listed in Table 2. The experimental results show that the total number of microdroplets, the CV values, and the number of effective microdroplets are stable, thereby meeting the requirements of the PCR instrument.

## 4. Conclusions

In order to alleviate the effects of undesirable surface wettability and pressure variation on the generation of droplets in COC-based devices, we have applied a simple surface modification procedure to hydrophobically modify the surfaces of COC-based microchannels for large-scale industrial production. In this work, the COC material was first plasma-cleaned and then soaked in FC-3283 solution containing 10% 1H, 1H, 2H, and 2H-perfluorooctane trichlorosilane for 2 h for the fluorination treatment, after which the related CA of water was increased from 94° to 114°. Moreover, after being left under an air atmosphere for 24 h, the COC substrate (following plasma cleaning) was then treated via fluorination. Consequently, it was found that there was no significant reduction in the water CA, which demonstrated that the fluorination treatment was still effective. The COC chip that did not undergo the fluorination treatment presented a weak bonding force between the substrate and the cover, and the sizes of the microdroplets could not reach the required level of 110 μm. Regarding the microdroplet generation test with the nonfluorinated COC chip, the sizes of the generated microdroplets exhibited an increasing trend for a given oil-phase pressure as the water-phase pressure continuously increased. Moreover, the water-phase flow at the cross junction showed an obvious extended tail. In other words, the uniformity of the microdroplets generated by the nonfluorinated COC chip would be affected by the irregular fluctuation of the water-phase flow in practical applications. On the other hand, the fluorination treatment for the COC chip after plasma cleaning can not only improve the bonding force but also remarkably improve the hysteresis of microdroplet generation at the cross junction. When the water-phase pressure was changed within a certain range, the sizes of the microdroplets generated by the fluorinated COC chip remained unchanged, endowing the chip with a certain anti-interference ability. The effects of the fluorination treatment tended to wane with the prolongation of the chips’ storage time. After 4 months of fluoride treatment, the chips were tested under the same experimental conditions, and the microdroplet sizes were generally reduced and thus failed to meet the requirements of practical use. The present study provides useful reference data for the mass production of COC-based microdroplet generation chips, and its results are of great significance with respect to the improvement of the detection accuracy of future digital PCR instruments.

## Figures and Tables

**Table 1:** Quantitative analysis of surface groups of COC, COC-O, and COC-O_10 min_-F.

Sample	Description		
	C-C/C-H (%)	C-O/C=O (%)	C-F/CF_2_/CF_3_ (%)
COC	87.6	12.4	/
COC-O	81.86	18.14	/
COC-O_10 min_-F	77.94	14.06	8.00

**Table 2:** Microdroplets tested by the flow digital PCR instrument (data extracted via software).

O/W-Parameter	Test Data			
	The Total Number of Microdroplets	CV Values	The Percentages of Effective Microdroplets	The Number of Effective Microdroplets
48/44 kPa	14,937	0.0291	90.55%	13,525
48/46 kPa	14,500	0.0286	92.41%	13,400
48/48 kPa	16,075	0.0287	92.14%	14,812

## Data Availability

Data will be available upon request.
